# Some mean convergence theorems for arrays of rowwise pairwise negative quadrant dependent random variables

**DOI:** 10.1186/s13660-018-1811-y

**Published:** 2018-08-23

**Authors:** Tapas K. Chandra, Deli Li, Andrew Rosalsky

**Affiliations:** 1 Applied Statistics Division, Indian Statistical Institute, Kolkata, India; 2 Department of Mathematical Sciences, Lakehead University, Thunder Bay, Canada; 3 Department of Statistics, University of Florida, Gainesville, USA

**Keywords:** 60F25, 60F99, 60F05, Array of rowwise pairwise negative quadrant dependent random variables, Pairwise independent random variables, Weighted averages, Degenerate mean convergence, Stochastic domination, Almost sure convergence

## Abstract

For arrays of rowwise pairwise negative quadrant dependent random variables, conditions are provided under which weighted averages converge in mean to 0 thereby extending a result of Chandra, and conditions are also provided under which normed and centered row sums converge in mean to 0. These results are new even if the random variables in each row of the array are independent. Examples are provided showing (i) that the results can fail if the rowwise pairwise negative quadrant dependent hypotheses are dispensed with, and (ii) that almost sure convergence does not necessarily hold.

## Introduction

For a sequence of independent and identically distributed (i.i.d.) random variables $\{X_{n}, n \geq 1 \}$ with $\mathbb{E}X_{1} = 0$, Pyke and Root [12] established the degenerate mean convergence law

1.1 $$ \frac{\sum_{j=1}^{n} X_{j}}{n} \stackrel{\mathscr{L}_{1}}{ \longrightarrow } 0. $$

A considerably simpler proof of the limit law (1.1) was obtained by Dharmadhikari [4] who did not refer to the Pyke and Root [12] article. Chandra [3] established the following more general result for mean convergence of weighted averages. Its proof is more natural, straightforward, and powerful than that of Dharmadhikari [4]. Chandra’s [3] method is novel in the sense that the level of truncation does not depend on *n* (the sample size), whereas Dharmadhikari [4] used the truncation level $\sqrt{n}$. The limit law (1.1) is obtained immediately from the Chandra [3] result by taking $a_{n,j} = n^{-1}$, $1 \leq j \leq n$, $n \geq 1$.

### Theorem 1.1

(Chandra [3], Theorem 1)

*Let* $\{X_{n}, n \geq 1 \}$ *be a sequence of pairwise i*.*i*.*d*. *random variables with* $\mathbb{E}X_{1} = 0$, *and let* $\{a_{n,j}, 1 \leq j \leq n, n \geq 1 \}$ *be a triangular array of constants such that*

$$ \sup_{n \geq 1} \sum_{j=1}^{n} \vert a_{n,j} \vert < \infty \quad \textit{and}\quad \lim _{n \rightarrow \infty } \sum_{j=1}^{n} a_{n,j}^{2} = 0. $$

*Then*

$$ \sum_{j=1}^{n} a_{n,j}X_{j} \stackrel{\mathscr{L}_{1}}{\longrightarrow } 0. $$

In the current work, we extend in Theorems 3.1 and 3.2 this degenerate mean convergence theorem of Chandra [3] in two directions:

(i) Our results pertain to weighted averages either from an *array* of random variables whose *n*th row is comprised of $k_{n}$ pairwise negative quadrant dependent random variables, $n \geq 1$ (Theorem 3.1) or from an *array* of random variables whose *n*th row is comprised of $k_{n}$ pairwise independent random variables, $n \geq 1$ (Theorem 3.2). No independence or dependence conditions are imposed between the random variables from different rows of the arrays. The Chandra [3] result considered weighted averages from a *sequence* of pairwise i.i.d. random variables.
(ii) The random variables that we consider are assumed to be stochastically dominated by a random variable which is a weaker assumption than the assumption of Chandra [3] that the random variables are identically distributed.

The third main result (Theorem 3.3) establishes for an array of random variables whose *n*th row is comprised of $k_{n}$ pairwise negative quandrant dependent random variables, $n \geq 1$ a degenerate mean convergence result for normed and centered row sums. In contradistinction to Theorems 3.1 and 3.2, weighted averages and stochastic domination play no role in Theorem 3.3. As in Theorems 3.1 and 3.2, no independence or dependence conditions are imposed between the random variables from different rows of the array in Theorem 3.3.

### Definition 1.1

A finite set of random variables $\{X_{1}, \ldots, X_{N} \}$ is said to be *pairwise negative quadrant dependent* (PNQD) if for all $i, j \in \{1, \ldots, N\}$ ($i \neq j$) and all $x, y \in \mathbb{R}$,

1.2 $$ \mathbb{P} ( X_{i} \leq x, X_{j} \leq y ) \leq \mathbb{P} ( X_{i} \leq x ) \mathbb{P} ( X_{j} \leq y ). $$

It is of course immediate that if $X_{1}, \ldots, X_{N} $ are pairwise independent (*a fortiori*, independent) random variables, then $\{X_{1}, \ldots, X_{N} \}$ is PNQD.

In many stochastic models, the classical assumption of independence among the random variables in the model is not a reasonable one; the random variable may be “repelling” in the sense that small values of any of the random variables increase the probability that the other random variables are large. Thus an assumption of some type of negative dependence is often more suitable. Pemantle [11] prepared an excellent survey on a general “theory of negative dependence”.

The choice of the adjective “negative” in the definition of PNQD random variables is due to the fact that (1.2) is equivalent to

$$ \mathbb{P} ( X_{j} > y \mid X_{i} \leq x ) \geq \mathbb{P} ( X_{j} > y ) $$

provided $\mathbb{P} ( X_{i} \leq x ) > 0$.

A collection of *N* PNQD random variables arises by sampling without replacement from a set of $N \geq 2$ real numbers (see, e.g., Bozorgnia et al. [2]). Li et al. [7] showed that for every set of $N \geq 2$ continuous distribution functions $\{F_{1}, \ldots, F_{N} \}$, there exists a set of PNQD random variables $\{X_{1}, \ldots, X_{N} \}$ such that the distribution function of $X_{j}$ is $F_{j}$, $1 \leq j \leq N$ and such that for all $j \in \{1, \ldots, N-1 \}$, $X_{j}$ and $X_{j+1}$ are not independent.

An array of random variables $\{X_{n,j}, 1 \leq j \leq k_{n}, n \geq 1 \}$ is said to be *rowwise* PNQD if for each $n \geq 1$, the set of random variables $\{X_{n,j}, 1 \leq j \leq k_{n} \}$ is PNQD. There is interesting literature of investigation on the strong law of large numbers problem for row sums of rowwise PNQD arrays; see the discussion in Li et al. [7].

### Definition 1.2

An array of random variables $\{X_{n,j}, 1 \leq j \leq k_{n}, n \geq 1 \}$ is said to be *stochastically dominated* by a random variable *X* if there exists a constant *D* such that

1.3 $$ \mathbb{P} \bigl( \vert X_{n,j} \vert > x \bigr) \leq D \mathbb{P} \bigl(\vert DX \vert > x \bigr), \quad x \geq 0, 1 \leq j \leq k_{n}, n \geq 1. $$

### Remark 1.1

Condition (1.3) is, of course, automatic with $X = X_{1,1}$ and $D = 1$ if the array $\{X_{n,j}, 1 \leq j \leq k _{n}, n \geq 1 \}$ consists of identically distributed random variables.

## Preliminary lemmas

Three lemmas will now be stated. Lemmas 2.1, 2.2, and 2.3 are used in the proof of Theorem 3.1, Lemma 2.3 is used in the proof of Theorem 3.2, and Lemmas 2.1 and 2.2 are used in the proof of Theorem 3.3.

Lemma 2.1 follows from Lemma 1 of Lehmann [6]; see Matuła [8] for a more direct proof.

### Lemma 2.1

(Lehmann [6], Matuła [8])

*Let the set of random variables* $\{ X_{1}, \ldots, X_{N} \} $ *be PNQD*, *and for each* $j \in \{1, \ldots, N \}$, *let* $f_{j}: \mathbb{R} \rightarrow \mathbb{R}$. *If the functions* $f_{1}, \ldots, f_{N}$ *are all nondecreasing or all nonincreasing*, *then the set of random variables* $\{ f_{1}(X _{1}), \ldots, f_{N}(X_{N}) \} $ *is PNQD*.

The next lemma is well known (see, e.g., Patterson and Taylor [10]).

### Lemma 2.2

*Let the set of random variables* $\{X_{1}, \ldots, X _{N} \}$ *be PNQD*. *Then*

$$ \operatorname{Var} \Biggl( \sum_{j=1}^{N} X_{j} \Biggr) \leq \sum_{j=1}^{N} \operatorname{Var} ( X_{j} ) . $$

The following lemma is essentially due to Adler et al. [1].

### Lemma 2.3

(Adler et al. [1])

*Let* $\{X_{n,j}, 1 \leq j \leq k_{n}, n \geq 1 \}$ *be an array of random variables which is stochastically dominated by a random variable* *X*, *and let* *D* *be as in* (1.3). *Then*

$$ \mathbb{E} \bigl( \vert X_{n,j} \vert I \bigl( \vert X_{n,j} \vert > x \bigr) \bigr) \leq D^{2} \mathbb{E} \bigl( \vert X \vert I \bigl(\vert DX \vert > x \bigr) \bigr), \quad x \geq 0, 1 \leq j \leq k_{n}, n \geq 1. $$

## Mainstream

The main results, Theorems 3.1–3.3, may now be established. These are new results even under the stronger hypothesis that the random variables in each row of the array are independent.

### Theorem 3.1

*Let* $\{X_{n,j}, 1 \leq j \leq k_{n}, n \geq 1 \}$ *be an array of rowwise PNQD mean* 0 *random variables which is stochastically dominated by a random variable* *X* *with* $\mathbb{E}\vert X \vert < \infty $. *Let* $\{a_{n,j}, 1 \leq j \leq k_{n}, n \geq 1 \}$ *be an array of constants such that*

3.1 $$ \textit{for each } n \geq 1, \textit{ either } \min _{1 \leq j \leq k_{n}} a_{n,j} \geq 0 \quad \textit{or}\quad \max _{1 \leq j \leq k_{n}} a_{n,j} \leq 0 $$

*and*

3.2 $$ \sup_{n \geq 1} \sum_{j=1}^{k_{n}} \vert a_{n,j} \vert < \infty \quad \textit{and} \quad\lim _{n \rightarrow \infty } \sum_{j=1}^{k_{n}} a_{n,j}^{2} = 0. $$

*Then*

3.3 $$ \sum_{j=1}^{k_{n}} a_{n,j} X_{n,j} \stackrel{\mathscr{L}_{1}}{ \longrightarrow } 0 $$

*and*, a fortiori,

$$ \sum_{j=1}^{k_{n}} a_{n,j} X_{n,j} \stackrel{\mathbb{P}}{\longrightarrow } 0. $$

### Proof

Let $\epsilon > 0$ be arbitrary. Set $C = \sup_{n \geq 1} \sum_{j=1}^{k_{n}} \vert a_{n,j} \vert $. Let $D < \infty $ be as in (1.3). Since $\mathbb{E}\vert X \vert < \infty $, we can choose $A_{\epsilon } \in (0, \infty )$ such that

$$ 2CD^{2} \mathbb{E} \bigl( \vert X \vert I \bigl(D\vert X \vert > A_{\epsilon } \bigr) \bigr) \leq \frac{ \epsilon }{2}\quad \mbox{and}\quad 2CDA_{\epsilon } \mathbb{P} \bigl( \vert DX \vert > A _{\epsilon }\bigr) \leq \frac{\epsilon }{2}. $$

Let

$$ Y_{n,j} = X_{n,j} I \bigl( \vert X_{n,j} \vert \leq A_{\epsilon } \bigr) + A_{ \epsilon } I \bigl( \vert X_{n,j} \vert > A_{\epsilon } \bigr) - A_{\epsilon } I \bigl( \vert X_{n,j} \vert < -A_{\epsilon } \bigr) , \quad 1 \leq j \leq k_{n}, n \geq 1 $$

and

$$ Z_{n,j} = X_{n,j} I \bigl( \vert X_{n,j} \vert > A_{\epsilon } \bigr) - A_{ \epsilon } I \bigl( \vert X_{n,j} \vert > A_{\epsilon } \bigr) + A_{\epsilon } I \bigl( \vert X_{n,j} \vert < -A_{\epsilon } \bigr) , \quad 1 \leq j \leq k_{n}, n \geq 1. $$

Then

$$ X_{n,j} = Y_{n,j} + Z_{n,j}\quad \mbox{and}\quad \mathbb{E}Y_{n,j} + \mathbb{E}Z_{n,j} = \mathbb{E}X_{n,j} = 0, \quad 1 \leq j \leq k_{n}, n \geq 1, $$

and so

$$ X_{n,j} = Y_{n,j} - \mathbb{E}Y_{n,j} + Z_{n,j} - \mathbb{E}Z_{n,j}, \quad 1 \leq j \leq k_{n},n \geq 1. $$

It follows from Lemma 2.1 that $\{Y_{n,j}, 1 \leq j \leq k_{n}, n \geq 1 \}$ is an array of rowwise PNQD random variables. Again by Lemma 2.1, (3.1) ensures that $\{a_{n,j}Y_{n,j}, 1 \leq j \leq k_{n}, n \geq 1 \}$ is an array of rowwise PNQD random variables. Note that $\vert Y_{n,j} \vert \leq A_{\epsilon }$, $1 \leq j \leq k_{n}$, $n \geq 1$. Thus, for $n \geq 1$, by Lemma 2.2

$$\begin{aligned} \mathbb{E} \Biggl( \sum_{j=1}^{k_{n}} a_{n,j} ( Y_{n,j} -\mathbb{E}Y_{n,j} ) \Biggr) ^{2} &=\operatorname{Var} \Biggl( \sum_{j=1}^{k_{n}} a_{n,j} Y_{n,j} \Biggr) \\ & \leq \sum_{j=1}^{k_{n}} a_{n,j}^{2} \operatorname{Var} ( Y_{n,j} ) \leq \sum_{j=1}^{k_{n}} a_{n,j}^{2} \mathbb{E} Y_{n,j}^{2} \leq A_{\epsilon }^{2}\sum_{j=1}^{k_{n}} a_{n,j}^{2} \\ &\rightarrow 0 \end{aligned}$$

by the second half of (3.2). Thus

$$ \sum_{j=1}^{k_{n}} a_{n,j} ( Y_{n,j} - \mathbb{E}Y_{n,j} ) \stackrel{ \mathscr{L}_{2}}{\longrightarrow } 0 $$

and, *a fortiori*,

3.4 $$ \sum_{j=1}^{k_{n}} a_{n,j} ( Y_{n,j} - \mathbb{E}Y_{n,j} ) \stackrel{ \mathscr{L}_{1}}{\longrightarrow } 0. $$

Next, for $n \geq 1$, by Lemma 2.3 and (1.3)

3.5 $$\begin{aligned} \mathbb{E} \Biggl\vert \sum_{j=1}^{k_{n}} a_{n,j} ( Z_{n,j} - \mathbb{E}Z_{n,j} ) \Biggr\vert & \leq 2\mathbb{E} \sum_{j=1}^{k_{n}} \vert a_{n,j} \vert \mathbb{E}\vert Z_{n,j} \vert \\ & \leq 2 \sum_{j=1}^{k_{n}} \vert a_{n,j} \vert \bigl( \mathbb{E} \bigl( \vert X_{n,j} \vert I \bigl(\vert X_{n,j} \vert > A_{\epsilon } \bigr) \bigr) + A_{\epsilon } \mathbb{P} \bigl( \vert X_{n,j} \vert > A_{\epsilon } \bigr) \bigr) \\ & \leq 2 \sum_{j=1}^{k_{n}} \vert a_{n,j} \vert \bigl( D^{2} \mathbb{E} \bigl( \vert X \vert I \bigl(\vert DX \vert > A_{\epsilon } \bigr) \bigr) + DA_{\epsilon } \mathbb{P} \bigl( \vert DX \vert > A _{\epsilon } \bigr) \bigr) \\ & \leq 2CD^{2} \mathbb{E} \bigl( \vert X \vert I \bigl(\vert DX \vert > A_{\epsilon } \bigr) \bigr) + 2CDA _{\epsilon } \mathbb{P} \bigl( \vert DX \vert > A_{\epsilon } \bigr) \\ & \leq \frac{\epsilon }{2} + \frac{\epsilon }{2} = \epsilon \end{aligned}$$

by the choice of $A_{\epsilon }$.

Combining (3.4) and (3.5) yields

$$\begin{aligned} \limsup_{n \rightarrow \infty } \mathbb{E} \Biggl\vert \sum _{j=1}^{k_{n}}a_{n,j} X_{n,j} \Biggr\vert & = \limsup_{n \rightarrow \infty } \mathbb{E} \Biggl\vert \sum _{j=1}^{k_{n}} a _{n,j} ( Y_{n,j} - \mathbb{E}Y_{n,j} + Z_{n,j} - \mathbb{E}Z_{n,j} ) \Biggr\vert \\ & \leq \limsup_{n \rightarrow \infty } \Biggl( \mathbb{E} \Biggl\vert \sum _{j=1}^{k _{n}} a_{n,j} ( Y_{n,j} - \mathbb{E}Y_{n,j} ) \Biggr\vert + \mathbb{E} \Biggl\vert \sum_{j=1}^{k_{n}} a_{n,j} ( Z_{n,j} - \mathbb{E}Z_{n,j} ) \Biggr\vert \Biggr) \\ &\leq \epsilon. \end{aligned}$$

Since $\epsilon > 0$ is arbitrary,

$$ \lim_{n \rightarrow \infty } \mathbb{E} \Biggl\vert \sum _{j=1}^{k_{n}} a _{n,j} X_{n,j} \Biggr\vert = 0; $$

that is, (3.3) holds. □

### Remark 3.1

One of the reviewers so kindly called to our attention the article by Ordóñez Cabrera and Volodin [9] and suggested that we should provide a comparison between Theorem 3.1 above and Theorem 1 of that article. Both theorems are in the same spirit in that they both establish mean convergence for weighted averages from an array of rowwise PNQD mean 0 random variables. Ordóñez Cabrera and Volodin [9] introduced the following new integrability concept for an array of random variables $\{X_{n,j}, u_{n} \leq j \leq k_{n}, n \geq 1 \}$ which is weaker than several well-known integrability notions. The array of random variables is said to be *h-integrable* with respect to an array of constants $\{a_{n,j}, u_{n} \leq j \leq k _{n}, n \geq 1 \}$ if

$$ \sup_{n \geq 1} \sum_{j = u_{n}}^{k_{n}} \vert a_{n,j} \vert \mathbb{E} \vert X_{n,j} \vert < \infty \quad \mbox{and}\quad \lim_{n \rightarrow \infty } \sum _{j = u_{n}} ^{k_{n}} \vert a_{n,j} \vert \mathbb{E} \bigl( \vert X_{n,j} \vert I \bigl(\vert X_{n,j} \vert > h(n) \bigr) \bigr) = 0, $$

where $\{h(n), n \geq 1 \}$ is a sequence of constants with $0 < h(n) \uparrow \infty $. Ordóñez Cabrera and Volodin [9] established their Theorem 1 under an *h*-integrability assumption for the array. Suppose that $u_{n} = 1$, $n \geq 1$. It is clear that the stochastic domination condition in Theorem 3.1 is indeed a stronger condition than the array being *h*-integrable. However, Theorem 1 of Ordóñez Cabrera and Volodin [9] has the condition $\lim_{n \rightarrow \infty } h^{2}(n) \sum_{j=1}^{k_{n}} a_{n,j}^{2} = 0$ which is stronger than the condition $\lim_{n \rightarrow \infty } \sum_{j=1}^{k_{n}} a_{n,j}^{2} = 0$ in (3.2) of Theorem 3.1. Consequently, the two theorems being compared overlap with each other but neither theorem is contained in the other.

The next theorem is a version of Theorem 3.1 without assumption (3.1) for an array of random variables where the random variables in each row of the array are pairwise independent (which is a stronger assumption than the array being rowwise PNQD).

### Theorem 3.2

*Let* $\{X_{n,j}, 1 \leq j \leq k_{n}, n \geq 1 \}$ *be an array of mean* 0 *random variables such that*, *for each* $n \geq 1$, *the random variables* $X_{n,j}, 1 \leq j \leq k_{n}$ *are pairwise independent*. *Suppose that the array* $\{X_{n,j}, 1 \leq j \leq k_{n}, n \geq 1 \}$ *is stochastically dominated by a random variable* *X* *with* $\mathbb{E}\vert X \vert < \infty $. *Let* $\{a_{n,j}, 1 \leq j \leq k_{n}, n \geq 1 \}$ *be an array of constants such that*

$$ \sup_{n \geq 1} \sum_{j=1}^{k_{n}} \vert a_{n,j} \vert < \infty \quad \textit{and}\quad \lim _{n \rightarrow \infty } \sum_{j=1}^{k_{n}} a_{n,j} ^{2} = 0. $$

*Then*

$$ \sum_{j=1}^{k_{n}} a_{n,j} X_{n,j} \stackrel{\mathscr{L}_{1}}{\longrightarrow } 0 $$

*and*, a fortiori,

$$ \sum_{j=1}^{k_{n}} a_{n,j} X_{n,j} \stackrel{\mathbb{P}}{\longrightarrow } 0. $$

### Proof

Let $\epsilon > 0$ be arbitrary, and let *C*, *D*, $A_{\epsilon }$, $Y_{n,j}$, and $Z_{n,j}$, $1 \leq j \leq k_{n}$, $n \geq 1$ be as in the proof of Theorem 3.1. The pairwise independence assumption ensures that

$$ \operatorname{Var} \Biggl( \sum_{j=1}^{k_{n}} a_{n,j}Y_{n,j} \Biggr) = \sum_{j=1} ^{k_{n}} a_{n,j}^{2} \operatorname{Var} ( Y_{n,j} ) , \quad n \geq 1, $$

and (3.4) follows arguing as in the proof of Theorem 3.1. Moreover, (3.5) holds by the same argument as in the proof of Theorem 3.1. The rest of the proof is identical to that in Theorem 3.1. □

### Remark 3.2

The cited result of Chandra [3] follows immediately from Theorem 3.2 by taking $k_{n} = n$, $n \geq 1$ and $X_{n,j} = X_{j}$, $1 \leq j \leq n$, $n \geq 1$.

### Remark 3.3

If the rowwise PNQD hypothesis in Theorem 3.1 is dispensed with, then the theorem can fail. To see this, let *X* be a nondegenerate mean 0 random variable, let $k_{n} = n$, $n \geq 1$, and let

$$ X_{n,j} = X\quad \mbox{and}\quad a_{n,j} = n^{-1}, \quad 1 \leq j \leq n,n \geq 1. $$

Then $\{X_{n,j}, 1 \leq j \leq n, n \geq 1\}$ is not an array of PNQD random variables, (3.1) and (3.2) hold, but

$$ \sum_{j=1}^{k_{n}} a_{n,j}X_{n,j} = X \stackrel{\mathscr{L}_{1}}{ \not \longrightarrow } 0. $$

This same example shows that Theorem 3.2 can fail without the pairwise independent hypothesis.

We now show via an example that the hypotheses to Theorems 3.1 and 3.2 do not necessarily ensure that $\sum_{j=1}^{k_{n}} a_{n,j}X_{n,j} \longrightarrow 0$ almost surely (a.s.).

### Example 3.1

Let $\{X_{n}, n \geq 1 \}$ be a sequence of i.i.d. random variables with $\mathbb{E}X_{1} = 0$ and $\mathbb{E}\vert X_{1} \vert ^{p} = \infty $ for some $p > 1$. Set $k_{n} = n$, $n \geq 1$, $X_{n,j} = X _{j}$, $1 \leq j \leq k_{n}$, $n \geq 1$, and

$$ a_{1,1} = 1, \qquad a_{n,j} = \textstyle\begin{cases} 0, & 1 \leq j \leq n, \\ n^{-1/p}, & j = n, \end{cases}\displaystyle \quad n\geq 2. $$

Then (3.2) holds since

$$ \sup_{n \geq 1} \sum_{j=1}^{k_{n}} \vert a_{n,j} \vert = \sup_{n \geq 1} n^{-1/p} = 1 < \infty $$

and

$$ \lim_{n \rightarrow \infty } \sum_{j=1}^{k_{n}} a_{n,j}^{2} = \lim_{n \rightarrow \infty } n^{-2/p} = 0. $$

All of the hypotheses of Theorems 3.1 and 3.2 are satisfied and hence (3.3) holds.

Note that $\{ \sum_{j=1}^{k_{n}} a_{n,j}X_{n,j} = n^{-1/p}X_{n}, n \geq 1 \} $ is a sequence of independent random variables. Now, for arbitrary $M \geq 1$,

$$ \sum_{n=1}^{\infty } \mathbb{P} \Biggl( \Biggl\vert \sum_{j=1}^{k_{n}} a _{n,j} X_{n,j} \Biggr\vert > M \Biggr) = \sum_{n=1}^{\infty } \mathbb{P} \bigl( \bigl\vert n^{-1/p}X_{n} \bigr\vert > M \bigr) = \sum_{n=1}^{\infty } \mathbb{P} \biggl( \frac{\vert X_{1} \vert }{M} > n^{1/p} \biggr) = \infty $$

since $\mathbb{E}\vert X_{1} \vert ^{p} = \infty $. Then by the second Borel–Cantelli lemma,

$$ \mathbb{P} \Biggl( \Biggl\vert \sum_{j=1}^{k_{n}} a_{n,j} X_{n,j} \Biggr\vert > M \mbox{ i.o. } (n) \Biggr) = 1, $$

and so

$$\begin{array}{rl} P\left(\underset{n\to \infty }{lim sup}|\sum_{j=1}^{k_{n}}a_{n,j}X_{n,j}|=\infty \right) & =P\left(\overset{\infty }{\underset{M=1}{⋂}}\left\{\underset{n\to \infty }{lim sup}|\sum_{j=1}^{k_{n}}a_{n,j}X_{n,j}|\geq M\right\}\right)\\ & \geq P\left(\overset{\infty }{\underset{M=1}{⋂}}\left\{|\sum_{j=1}^{k_{n}}a_{n,j}X_{n,j}|>M\text{ i.o. }(n)\right\}\right)\\ & =1. \end{array}$$

Thus,

$$ \limsup_{n \rightarrow \infty } \Biggl\vert \sum_{j=1}^{k_{n}} a_{n,j} X _{n,j} \Biggr\vert = \infty \quad \mbox{a.s.} $$

and so $\sum_{j=1}^{k_{n}} a_{n,j} X_{n,j} \rightarrow 0$ a.s. fails.

We now establish Theorem 3.3. Throughout the rest of this section, for an array of random variables $\{ X_{n,j}, 1 \leq j \leq k_{n}, n \geq 1 \} $, let $S_{n} = \sum_{j=1}^{k_{n}} X_{n,j}$, $n \geq 1$.

### Theorem 3.3

*Let* $\{ X_{n,j}, 1 \leq j \leq k_{n}, n \geq 1 \} $ *be an array of rowwise PNQD* $\mathscr{L}_{1}$ *random variables*. *Let* $g:[0, \infty ) \rightarrow [0, \infty )$ *be a continuous function with*

$$ g(0) = 0\quad \textit{and}\quad \frac{g^{2}(v)}{v} \uparrow \infty \quad \textit{as }0 < v \uparrow \infty . $$

*Let* $\{b_{n}, n \geq 1 \}$ *be a sequence of positive constants with* $b_{n} \uparrow \infty $ *and suppose that there exists a sequence of positive constants* $\{\alpha_{n}, n \geq 1 \}$ *such that*

3.6 $$ g(v) - g(b_{n}) \leq \alpha_{n} (v - b_{n})\quad \textit{for all } v > b_{n}\textit{ and } n \geq 1. $$

*Set*

$$ V_{n,j} = g^{-1} \bigl( \vert X_{n,j} \vert \bigr) ,\quad 1 \leq j \leq k_{n},n \geq 1 $$

*and assume that* $\mathbb{E}V_{n,j} < \infty $, $1 \leq j \leq k_{n}$, $n \geq 1$. *Let* $\{d_{n}, n \geq 1\}$ *be a sequence of positive constants and suppose for some sequence of positive constants* $\{c_{n}, n \geq 1 \}$ *with* $c_{n} < b_{n}$, $n \geq 1$ *that*

3.7 $$\begin{aligned}& \frac{\alpha_{n}}{d_{n}} \sum_{j=1}^{k_{n}} \mathbb{E} \bigl( ( V _{n,j} - b_{n} ) I ( V_{n,j} > b_{n} ) \bigr) \rightarrow 0, \end{aligned}$$

3.8 $$\begin{aligned}& \frac{g^{2}(b_{n})}{d_{n}^{2}b_{n}} \sum_{j=1}^{k_{n}} \mathbb{E} \bigl( V_{n,j} I ( V_{n,j} > c_{n} ) \bigr) \rightarrow 0, \end{aligned}$$

3.9 $$\begin{aligned}& \frac{g^{2}(b_{n})}{d_{n}^{2}b_{n}} \sum_{j=1}^{k_{n}} \mathbb{E} V _{n,j} = \mathit{O}(1), \end{aligned}$$

3.10 $$\begin{aligned}& \frac{g^{2}(b_{n})}{d_{n}^{2}} \sum_{j=1}^{k_{n}} \mathbb{P} ( V _{n,j} > b_{n} ) \rightarrow 0, \end{aligned}$$

*and*

3.11 $$ \frac{g^{2}(c_{n})}{c_{n}} = \mathit{o} \biggl( \frac{g^{2}(b_{n})}{b _{n}} \biggr) . $$

*Then*

3.12 $$ \frac{S_{n} - \mathbb{E}S_{n}}{d_{n}} \stackrel{\mathscr{L}_{1}}{ \longrightarrow } 0 $$

*and*, a fortiori,

$$ \frac{S_{n} - \mathbb{E}S_{n}}{d_{n}} \stackrel{\mathbb{P}}{\longrightarrow } 0. $$

### Proof

For $1 \leq j \leq k_{n}$ and $n \geq 1$, set

$$ Y_{n,j} = X_{n,j}I \bigl( \vert X_{n,j} \vert \leq g(b_{n}) \bigr) + g(b_{n})I \bigl( X_{n,j} > g(b_{n}) \bigr) - g(b_{n})I \bigl( X _{n,j} < - g(b_{n}) \bigr) $$

and

$$ Z_{n,j} = \bigl( X_{n,j} - g(b_{n}) \bigr) I \bigl( X_{n,j} > g(b_{n}) \bigr) + \bigl( X_{n,j} + g(b_{n}) \bigr) I \bigl( X_{n,j} < - g(b _{n}) \bigr) . $$

Then $Y_{n,j} + Z_{n,j} = X_{n,j}$, $1 \leq j \leq k_{n}$, $n \geq 1$. Set $T_{n} = \sum_{j=1}^{k_{n}}Y_{n,j}$, $n \geq 1$. We will show that

3.13 $$ \frac{\sum_{j=1}^{k_{n}} \mathbb{E}\vert Z_{n,j} \vert }{d_{n}} \rightarrow 0 $$

and

3.14 $$ \frac{T_{n} - \mathbb{E}T_{n}}{d_{n}} \stackrel{\mathscr{L}_{1}}{ \longrightarrow } 0. $$

To prove (3.13), note that for $1 \leq j \leq k_{n}$ and $n \geq 1$,

$$\begin{aligned} \vert Z_{n,j} \vert &= \bigl( X_{n,j} - g(b_{n}) \bigr) I \bigl( X_{n,j} > g(b_{n}) \bigr) + \bigl( -X_{n,j} - g(b_{n}) \bigr) I \bigl( X_{n,j} < - g(b_{n}) \bigr) \\ &= \bigl( \vert X_{n,j} \vert - g(b_{n}) \bigr) I \bigl( X_{n,j} > g(b _{n}) \bigr) + \bigl( \vert X_{n,j} \vert - g(b_{n}) \bigr) I \bigl( X_{n,j} < - g(b_{n}) \bigr) \\ &= \bigl( \vert X_{n,j} \vert - g(b_{n}) \bigr) I \bigl( \vert X_{n,j} \vert > g(b_{n}) \bigr) \\ &= \bigl( g ( V_{n,j} ) - g(b_{n}) \bigr) I \bigl( g ( V _{n,j} ) > g(b_{n}) \bigr) \\ &= \bigl( g ( V_{n,j} ) - g(b_{n}) \bigr) I ( V_{n,j} > b _{n} ) \\ & \leq \alpha_{n} ( V_{n,j} - b_{n} ) I ( V_{n,j} > b_{n} )\quad \bigl(\mbox{by }(3.6) \bigr), \end{aligned}$$

and hence

$$ \frac{1}{d_{n}} \sum_{j=1}^{k_{n}} \mathbb{E}\vert Z_{n,j} \vert \leq \frac{\alpha_{n}}{d_{n}} \sum _{j=1}^{k_{n}} \mathbb{E} \bigl( ( V_{n,j} - b_{n} ) I ( V_{n,j} > b_{n} ) \bigr) \rightarrow 0 $$

by (3.7) proving (3.13).

To prove (3.14), note that for $1 \leq j \leq k_{n}$ and $n \geq 1$,

$$\begin{aligned} Y_{n,j}^{2} & = X_{n,j}^{2} I \bigl( \vert X_{n,j} \vert \leq g(b_{n}) \bigr) + g^{2}(b_{n}) I \bigl( \vert X_{n,j} \vert > g(b_{n}) \bigr) \\ & = g^{2}(V_{n,j}) I \bigl( g(V_{n,j}) \leq g(b_{n}) \bigr) + g^{2}(b_{n}) I \bigl( g(V_{n,j}) > g(b_{n}) \bigr) \\ & = g^{2}(V_{n,j}) I ( V_{n,j} \leq b_{n} ) + g^{2}(b_{n}) I ( V_{n,j} > b_{n} ) \\ & = \frac{g^{2}(V_{n,j})}{V_{n,j}} \cdot V_{n,j} I ( 0 < V_{n,j} \leq c_{n} ) + \frac{g^{2}(V_{n,j})}{V_{n,j}} \cdot V_{n,j} I ( c_{n} < V_{n,j} \leq b_{n} ) + g^{2}(b_{n}) I ( V_{n,j} > b_{n} ). \end{aligned}$$

Then for $n \geq 1$, since the set of random variables $\{Y_{n,j}, 1 \leq j \leq k_{n} \}$ is PNQD by Lemma 2.1,

$$\begin{aligned} &\mathbb{E} \biggl( \frac{T_{n} - \mathbb{E}T_{n}}{d_{n}} \biggr) ^{2} \\ &\quad \leq \frac{1}{d_{n}^{2}} \sum_{j=1}^{k_{n}} \operatorname{Var} ( Y_{n,j} ) \quad (\mbox{by Lemma}~2.2) \\ &\quad \leq \frac{1}{d_{n}^{2}} \sum_{j=1}^{k_{n}}\mathbb{E}Y_{n,j}^{2} \\ &\quad = \frac{1}{d_{n}^{2}} \Biggl( \sum_{j=1}^{k_{n}}\mathbb{E} \biggl( \frac{g^{2}(V_{n,j})}{V_{n,j}} \cdot V_{n,j} I ( 0 < V_{n,j} \leq c_{n} ) \biggr) \\ &\quad \quad {}+ \sum _{j=1}^{k_{n}} \mathbb{E} \biggl( \frac{g^{2}(V _{n,j})}{V_{n,j}} \cdot V_{n,j} I ( c_{n} < V_{n,j} \leq b_{n} ) \biggr) \\ &\quad \quad {}+ \sum_{j=1}^{k_{n}} \mathbb{E} \bigl( g^{2}(b_{n})I ( V_{n,j} > b_{n} ) \bigr) \Biggr) \\ &\quad \leq \frac{1}{d_{n}^{2}} \Biggl( \frac{g^{2}(c_{n})}{c_{n}} \sum _{j=1} ^{k_{n}} \mathbb{E}V_{n,j} + \frac{g^{2}(b_{n})}{b_{n}} \sum_{j=1} ^{k_{n}} \mathbb{E} \bigl( V_{n,j} I ( V_{n,j} > c_{n} ) \bigr) + g^{2}(b_{n}) \sum_{j=1}^{k_{n}} \mathbb{P} ( V_{n,j} > b _{n} ) \Biggr) \\ &\quad = \frac{1}{d_{n}^{2}} \Biggl( \frac{g^{2}(b_{n})}{b_{n}} \cdot {\mathit{o}}(1) \sum _{j=1}^{k_{n}} \mathbb{E}V_{n,j} \\ &\quad \quad {} + \frac{g^{2}(b_{n})}{b_{n}} \sum_{j=1}^{k_{n}} \mathbb{E} \bigl( V_{n,j} I ( V_{n,j} > c_{n} ) \bigr) + g^{2}(b_{n}) \sum_{j=1}^{k_{n}} \mathbb{P} ( V _{n,j} > b_{n} ) \Biggr) \quad \bigl(\mbox{by }(3.11) \bigr) \\ &\quad = \frac{g^{2}(b_{n})}{d_{n}^{2}b_{n}} \Biggl( \sum_{j=1}^{k_{n}}\mathbb{E}V_{n,j} \Biggr) \cdot {\mathit{o}}(1) + \frac{g^{2}(b_{n})}{d _{n}^{2}b_{n}} \sum_{j=1}^{k_{n}} \mathbb{E} \bigl(V_{n,j} I ( V _{n,j} > c_{n} ) \bigr) + \frac{g^{2}(b_{n})}{d_{n}^{2}} \sum_{j=1}^{k_{n}} \mathbb{P} (V_{n,j} > b_{n} ) \\ & \quad = \mathit{o}(1) \quad \bigl(\mbox{by }(3.9), (3.8),\mbox{ and } (3.10) \bigr). \end{aligned}$$

Thus

$$ \frac{T_{n} - \mathbb{E}T_{n}}{d_{n}} \stackrel{\mathscr{L}_{2}}{ \longrightarrow } 0 $$

and hence (3.14) holds.

Finally, note that for $n \geq 1$,

3.15 $$\begin{aligned} \frac{S_{n} - \mathbb{E}S_{n}}{d_{n}} & = \frac{\sum_{j=1}^{k_{n}}Y_{n,j} + \sum_{j=1}^{k_{n}}Z_{n,j} - \sum_{j=1}^{k_{n}} \mathbb{E}Y_{n,j} - \sum_{j=1}^{k_{n}} \mathbb{E}Z_{n,j} }{d_{n}} \\ & = \frac{\sum_{j=1}^{k_{n}}Z_{n,j} - \sum_{j=1}^{k_{n}} \mathbb{E}Z_{n,j} }{d_{n}} + \frac{T_{n} - \mathbb{E}T_{n}}{d_{n}}. \end{aligned}$$

Now it follows from (3.13) that

3.16 $$ \mathbb{E} \biggl\vert \frac{\sum_{j=1}^{k_{n}}Z_{n,j} - \sum_{j=1}^{k _{n}} \mathbb{E}Z_{n,j} }{d_{n}} \biggr\vert \leq \frac{2 \sum_{j=1}^{k _{n}} \mathbb{E}\vert Z_{n,j} \vert }{d_{n}} \rightarrow 0. $$

The conclusion (3.12) follows from (3.15), (3.16), and (3.14). □

### Corollary 3.1

*Let* $\{X_{n,j}, 1 \leq j \leq k_{n}, n \geq 1 \}$ *be a uniformly bounded array of rowwise PNQD random variables*. *Let* $\{b_{n}, n \geq 1 \}$ *be a sequence of constants with* $1 < b_{n} \uparrow \infty $. *Then*

3.17 $$ \frac{S_{n} - \mathbb{E}S_{n}}{\sqrt{k_{n} b_{n}}} \stackrel{ \mathscr{L}_{1}}{ \longrightarrow } 0. $$

### Proof

Let $d_{n} = \sqrt{k_{n}b_{n}}$, $n \geq 1$ and $c_{n} = \sqrt{b_{n}}$, $n \geq 1$. Let $g(v) = v$, $v \geq 0$ and $\alpha_{n} = 1$, $n \geq 1$. Set

$$ V_{n,j} = g^{-1} \bigl( \vert X_{n,j} \vert \bigr) = \vert X_{n,j} \vert , \quad 1 \leq j \leq k_{n}, n \geq 1. $$

Since the array is comprised of uniformly bounded random variables, conditions (3.7), (3.8), (3.9), and (3.10) hold. Moreover, (3.11) also holds since $c_{n} = \mathit{o} ( b_{n} ) $. The conclusion (3.17) follows from Theorem 3.3. □

### Remark 3.4

If the rowwise PNQD hypothesis in Theorem 3.3 or Corollary 3.1 is dispensed with, then those results can fail. To see this, let $\{k_{n}, n \geq 1 \}$ be a sequence of integers with $1 < k_{n} \uparrow \infty $, let *X* be a bounded nondegenerate random variable, and set $X_{n,j} = X$, $1 \leq j \leq k_{n}$, $n \geq 1$. Let $b_{n} = k_{n}$, $n \geq 1$. All of the conditions of Corollary 3.1 (hence of Theorem 3.3) are satisfied except for the rowwise PNQD hypothesis. The conclusions of Corollary 3.1 (hence of Theorem 3.3) fail since

$$ \frac{S_{n} - \mathbb{E}S_{n}}{\sqrt{k_{n} b_{n}}} = \frac{k_{n}(X - \mathbb{E}X)}{k_{n}} = X - \mathbb{E}X \stackrel{ \mathscr{L}_{1}}{ \not \longrightarrow } 0. $$

We now show via an example that the hypotheses of Corollary 3.1 (hence of Theorem 3.3) do not necessarily ensure that

3.18 $$ \frac{S_{n} - \mathbb{E}S_{n}}{\sqrt{k_{n} b_{n}}} \rightarrow 0\quad \mbox{a.s.} $$

### Example 3.2

Let $\{X_{n}, n \geq 1\}$ be a sequence of nondegenerate i.i.d. uniformly bounded random variables, and let

$$ k_{n} = n,\qquad b_{n} = \log \log \bigl(\max \{16, n\} \bigr),\qquad d_{n} = \sqrt{k _{n}b_{n}},\quad n \geq 1. $$

Let $X_{n,j} = X_{j}$, $1 \leq j \leq n$, $n \geq 1$. The hypotheses of Corollary 3.1 (hence of Theorem 3.3) are satisfied and so (3.12) and (3.17) hold. But by the Hartman and Wintner [5] law of the iterated logarithm,

$$ \limsup_{n \rightarrow \infty } \frac{S_{n} - \mathbb{E}S_{n}}{\sqrt{k _{n} b_{n}}} = \sqrt{2 \operatorname{Var}(X_{1})}\quad \mbox{a.s.} $$

and so (3.18) does not hold.

### Corollary 3.2

*Let* $\{X_{n,j}, 1 \leq j \leq n, n \geq 1 \}$ *be an array of identically distributed rowwise PNQD* $\mathscr{L}_{1}$ *random variables*, *and let* $\{b_{n}, n \geq 1 \}$ *be a sequence of constants with* $1 < b_{n} \uparrow \infty $. *If*

3.19 $$ \frac{\sqrt{n}}{\sqrt{b_{n}}} \mathbb{E} \bigl( \bigl(\vert X_{1,1} \vert - b _{n} \bigr) I \bigl(\vert X_{1,1} \vert > b_{n} \bigr) \bigr) \rightarrow 0, $$

*then*

3.20 $$ \frac{S_{n} - \mathbb{E}S_{n}}{\sqrt{n b_{n}}} \stackrel{ \mathscr{L}_{1}}{ \longrightarrow } 0. $$

### Proof

We will apply Theorem 3.3 with $g(v) = v$, $0 \leq v < \infty $ and

$$ k_{n} = n,\qquad \alpha_{n} = 1,\qquad c_{n} = \sqrt{b_{n}},\quad \mbox{and}\quad d _{n} = \sqrt{nb_{n}},\quad n \geq 1. $$

Then $c_{n} < b_{n}$, $n \geq 1$ and (3.6) and (3.11) are immediate. Condition (3.7) is the same as (3.19). Since $\mathbb{E}\vert X_{1,1} \vert < \infty $, conditions (3.8) and (3.9) are immediate. Condition (3.10) reduces to

$$ b_{n} \mathbb{P} \bigl( \vert X_{1,1} \vert > b_{n} \bigr) \rightarrow 0 $$

which holds since $\mathbb{E}\vert X_{1,1} \vert < \infty $. The conclusion (3.20) follows from Theorem 3.3. □

## Conclusions

For an array of rowwise PNQD random variables $\{X_{n,j}, 1 \leq j \leq k_{n}, n \geq 1 \}$, conditions are provided under which the following degenerate mean convergence laws hold:

(i) $$\sum_{j=1}^{k_{n}}a_{n,j}X_{n,j} \stackrel{\mathscr{L}_{1}}{\longrightarrow} 0, $$
  where $\mathbb{E}X_{n,j} = 0$, $1 \leq j \leq k_{n}$, $n \geq 1$, and $\{ a_{n,j}, 1 \leq j \leq k_{n}, n \geq 1 \} $ is an array of constants;
(ii) $$\frac{\sum_{j=1}^{k_{n}} ( X_{n,j} - \mathbb{E}X_{n,j} ) }{d_{n}} \stackrel{\mathscr{L}_{1}}{\longrightarrow } 0, $$
  where $\{d_{n}, n \geq 1 \}$ is a sequence of positive constants.

A version of the result in (i) is also obtained for an array of rowwise pairwise independent random variables and this result extends the result of Chandra [3]. Examples are provided showing that the above results can fail if the hypotheses are weakened and that a.s. convergence does not necessarily hold together with the $\mathscr{L}_{1}$ convergence.
